# Dual-drug delivery system based on cubosomes for glioblastoma therapy: electrochemical, structural and biological characterization

**DOI:** 10.1039/d6na00459h

**Published:** 2026-08-18

**Authors:** Ewelina Dąbrowska, Damian Gaweł, Marlena Godlewska, Ewa Gajda, Renata Bilewicz, Ewa Nazaruk

**Affiliations:** a Faculty of Chemistry, University of Warsaw Pasteura 1 Warsaw 02-093 Poland enaz@chem.uw.edu.pl; b Department of Cell Biology and Immunology, Centre of Postgraduate Medical Education Marymoncka 99/103 Warsaw 01-813 Poland

## Abstract

Monotherapy using a single drug exhibits significant limitations in the treatment of complex diseases, particularly cancer, where tumor heterogeneity often restricts therapeutic efficacy. Dual-drug delivery systems enable the co-administration of therapeutics with distinct and complementary mechanisms of action, offering a promising strategy to overcome these limitations. In this study, we developed a dual-drug delivery system based on lipid liquid-crystalline cubic phases (LCPs) co-loaded with temozolomide (TMZ) and doxorubicin (DOX). TMZ induces DNA alkylation, whereas DOX inhibits topoisomerase II, thereby reducing the likelihood of developing resistance by tumor. Electrochemical methods were employed to investigate drug release kinetics from mono- and dual-drug-loaded cubosomes and to detect any interactions between the drugs or with the lipid forming the carrier that affect the release behavior of each drug from the nanocarrier system. They also allowed monitoring of the hydrolysis of TMZ and proposal of the optimal ratio of drugs in the carrier, taking into account this degradation process. The simultaneous administration of TMZ and DOX may improve treatment efficacy through synergistic effects and permit dose reduction compared with monotherapy. The structural properties of the nanoparticles were characterized using small-angle X-ray scattering (SAXS) and dynamic light scattering (DLS). Biological evaluation demonstrated that combination treatment with a standard concentration of TMZ and a lower concentration of DOX resulted in improved therapeutic outcomes in A172 glioblastoma-derived and HeLa cell lines.

## Abstract 2

Co-delivery of chemo-drugs in cubosomes enhances anticancer efficacy, offering a promising strategy for cancer treatment.

## Graphical abstract

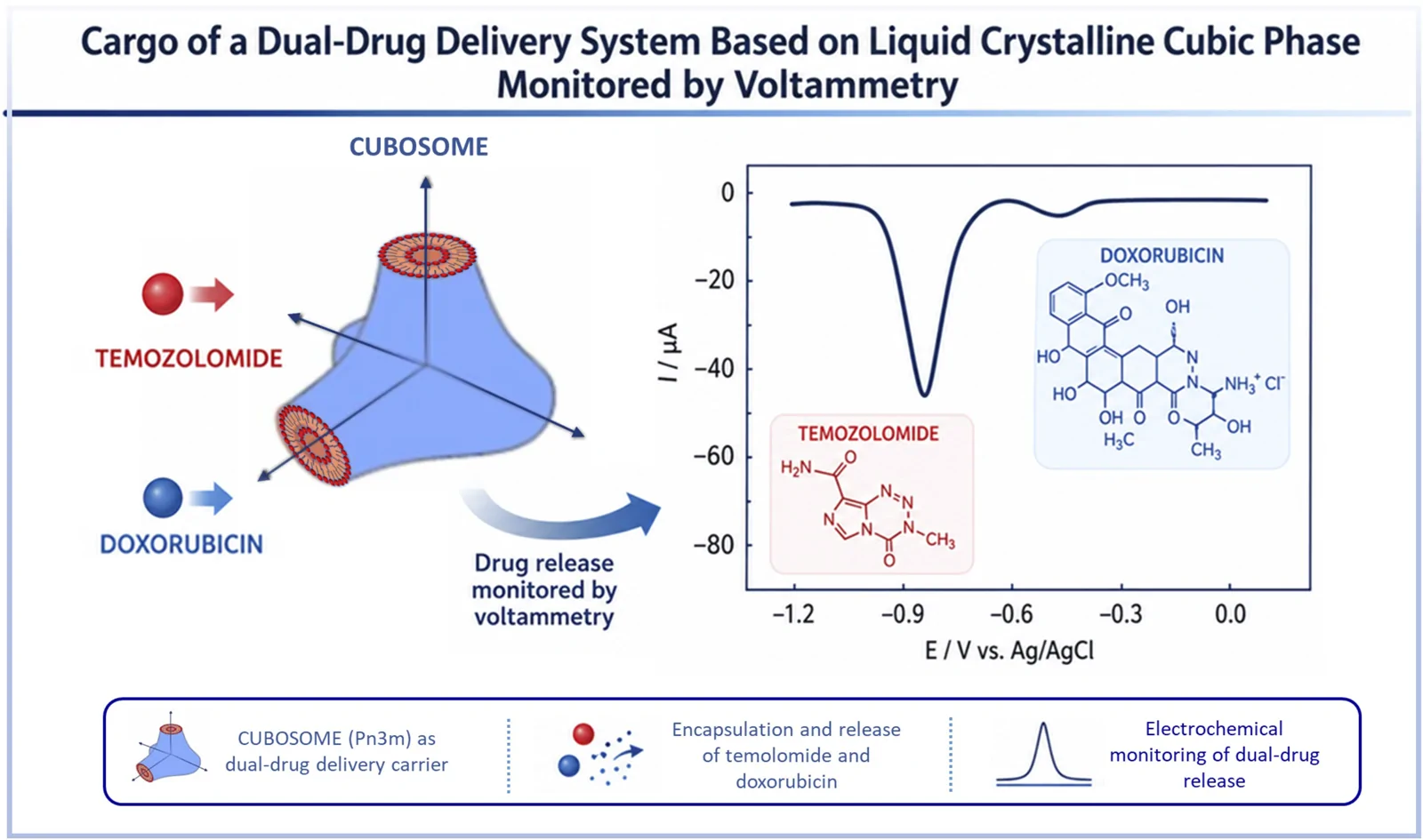

## 1 Introduction

Monotherapy using a single drug exhibits significant limitations in the treatment of complex diseases, particularly cancer, where tumor heterogeneity often restricts therapeutic efficacy. There is a need for alternative strategies, such as combination therapy, which uses existing drugs more effectively. Using a multi-drug delivery system is emerging as a strategy to co-deliver multiple drugs to the same site simultaneously. Combining different anticancer agents allows lower doses of each drug, targets multiple cancer pathways, and offers advantages including increased efficacy, reduced toxicity, and decreased risk of drug resistance. Dual-drug delivery systems enable the co-administration of therapeutics with distinct and complementary mechanisms of action, offering a promising strategy to overcome these limitations.^1,2^ Co-encapsulating two drugs in a single nanoformulation has been shown to reduce tumor growth by an additional 19% compared with combining two separately encapsulated nanomedicines.^2^

Self-assembled lipid-based liquid crystalline nanoparticles (LCNPs) have gained significant attention as multifunctional drug delivery systems. They can accommodate both lipophilic and hydrophilic drugs and may be able to release them in a controlled way. In drug delivery applications, inverted lipid structures are particularly appealing due to their stability in excess water. Moreover, the monoolein (MO)- and monopalmitolein (MP)-based systems are attractive because they form cubic phases within the physiological temperature range.^3^ Common inverse lipid architectures include lamellar, 2D hexagonal (H2), and complex 3D structures such as micellar cubic (I2) and bicontinuous cubic (V2) phases (primitive P, diamond D, and gyroid G). Lipid liquid-crystalline cubic phases (LCPs) consist of a curved bicontinuous lipid bilayer surrounding two interpenetrating, non-contacting aqueous channels and offer a large interfacial area (∼400 m^2^ g^−1^), making them promising drug carriers.^4–8^ Cubosomes have already been proven to be excellent drug delivery systems^9^ and have also been used for anticancer drugs,^10–12^ and co-delivery of two drugs loaded into cubosomes has been reported.^13,14^ According to the research findings, non-lamellar liquid crystal nanoparticles outperform liposomes in terms of cellular interactions and uptake efficiencies.^15^ Cubosomes, in particular, have attracted interest due to their ability to fuse with cell membranes, potentially overcoming endosomal escape barriers and enhancing cellular uptake.

Glioblastoma (GBM) is the most common primary brain tumor and is associated with a poor long-term prognosis. The current standard treatment includes surgical resection, radiotherapy, and chemotherapy with temozolomide (TMZ). TMZ is lipophilic, allowing it to cross the blood–brain barrier (BBB) and induce cell death in glioma cells. Although TMZ can cross the BBB, high systemic doses are often required, which may cause severe side effects.^16,17^ The active metabolite of TMZ, 5-(3-methyltriazen-1-yl)imidazole-4-carboxamide (MTIC), is hydrolyzed to methylate O6-guanine residues in DNA, resulting in DNA damage and GBM cell death.^18^ Recently, it was shown that TMZ/DOX dual therapy can both inhibit proliferation and enhance cytotoxicity in GBM cells. DOX was effective at doses up to four times lower than those of TMZ, and combination therapy achieved irreversible growth inhibition where monotherapy failed. The co-delivery of TMZ and DOX was shown to enhance therapeutic efficacy through synergistic action and allowed dose reduction when compared to monotherapy.^19^ DOX is a non-selective anthracycline antitumor antibiotic. The mechanism of action of DOX involves (i) disruption of DNA repair *via* topoisomerase II inhibition, leading to DNA fragmentation; (ii) intercalation into genomic and mitochondrial DNA, inhibiting transcription; and (iii) generation of iron-mediated quinone free radicals, causing structural damage and ultimately cell death.^20^ Both TMZ and DOX target distinct cancer pathways, helping to overcome resistance associated with individual agents. The conjugation of TMZ to DOX *via* an acylhydrazone linkage was proposed to form a double intercalating agent and add more possibility of DNA damage, since specific access to DNA in the nucleus is favored by the DOX moiety.^21^ Our approach does not require conjugation procedures, protects both drugs by encapsulating them in the cubosome structure, and allows them to perform their differing functions. Using reduced concentrations mitigates toxicity while maintaining high efficacy. The potent cytotoxicity of DOX, combined with TMZ, offers a promising approach to enhance tumor reduction and improve patient survival.^22^

Recently, we demonstrated that TMZ encapsulated in MP cubic phases shows promising therapeutic potential and outperforms the drug-loaded MO phase as a monodrug delivery system.^23^ Co-administration of miR-7-5p with TMZ has been suggested to reduce multidrug resistance and induce apoptosis.^24^ In the present study, we investigated the effects of TMZ combined with DOX, incorporated into MO- and MP-derived cubic phases, on the GBM (A172) and HeLa cell lines. The structural properties of the lipid nanoparticles were analyzed using small-angle X-ray scattering (SAXS) and dynamic light scattering (DLS). Electrochemical methods were employed to evaluate drug release from cubic phases and cubosomes and to monitor TMZ metabolites in aqueous solution, building on prior reports that its decomposition can be tracked electrochemically.^18,25^ Biological evaluation showed that combined treatment with a higher concentration of temozolomide (100 µM) and a lower concentration of doxorubicin (1.25 µM) resulted in improved therapeutic outcomes. Under these conditions, differential pulse voltammetry was used to characterize the drug release profile from the carrier.

## 2 Materials and chemicals

### 2.1 Chemicals and reagents

Monoolein (1-oleoyl-*rac*-glycerol; purity ≥ 99%), TMZ, dimethyl sulfoxide (DMSO), doxorubicin, and Pluronic F127 were purchased from Sigma-Aldrich (Sigma-Aldrich, USA). Monopalmitolein (1-(9*Z*-hexadecenoyl)-*rac*-glycerol) was purchased from Jena Bioscience (Jena Bioscience, Germany). MES buffer at pH 5.3 was used, prepared from 2-(*N*-morpholino)ethanesulfonic acid, purchased from Avantor Performance Materials Poland S.A. All solutions were prepared with Milli-Q water (18.2 MΩ cm^−1^; Millipore, USA).

### 2.2 Preparation of the monoolein and monopalmitolein mesophases

Cubic phases were prepared according to the phase diagrams of the monoolein : water and monopalmitolein : water systems.^5^ Non-doped cubic phases were obtained by mixing MO or MP with a pH 5.3 buffer at weight ratios of 60 : 40 and 50 : 50, respectively. Drug-loaded cubic phases containing DOX and TMZ at a 1 : 3 molar ratio were prepared by dissolving the drugs in a buffer solution (pH 5.3) and adding the solution to the melted lipid. Additional formulations with a DOX : TMZ molar ratio of 1 : 80 were also prepared and evaluated *in vitro* using A172 and HeLa cell lines. The chemical structures of the compounds are shown in Scheme S1.

MO cubosomes were prepared by mixing the lipid with Pluronic F127 at a mass ratio of 8 : 1. DOX dissolved in 200 µL of MES buffer (pH 5.3) and TMZ dissolved in 120 µL of DMSO were added to the melted MO (100 mg). The DOX : TMZ molar ratio was 1 : 80. Subsequently, MES buffer (pH 5.3) was added to obtain a total aqueous content of 1 mL. MP cubosomes were prepared analogously: the lipid was mixed with Pluronic F127 at the same 8 : 1 ratio, followed by the addition of identical amounts of DOX and TMZ and the use of the same buffer composition and dilution procedure.

### 2.3 Structural characterization of the monoolein and monopalmitolein mesophases

Small-angle X-ray scattering analysis was performed to investigate the internal structure and phase organization of the liquid crystalline systems. Measurements were conducted using a Bruker Nanostar instrument with Cu Kα radiation and a Vantec 2000 area detector. The recorded two-dimensional scattering images were radially averaged to obtain one-dimensional intensity profiles, *I*(*q*), where the scattering vector magnitude *q* was calculated according to *q* = (4π/*λ*)sin *θ*. The samples were transferred to 1.5 mm capillaries and equilibrated under ambient conditions for at least 12 h before data acquisition. All measurements were carried out at 25 °C. Data collection times were set to 5 h for dispersed nanoparticle systems and 10 min for bulk mesophases. Phase identification was performed by indexing diffraction peaks through assignment of Miller indices (*hkl*), based on the ratios of successive scattering vectors to the primary peak (*q*_0_). Distinct liquid-crystalline phases were identified according to the characteristic relationships between Bragg reflections observed in the SAXS patterns. Unit cell parameters were extracted from peak positions in the SAXS patterns and subsequently used to derive structural characteristics, including bilayer thickness and aqueous channel dimensions, following the methodology described in ref. 26.

The hydrodynamic size distribution and polydispersity of nanoparticles were determined by dynamic light scattering using a Zetasizer Nano ZS (Malvern, UK) at 25 °C, assuming a water viscosity. Measurements were performed in triplicate, and refractive indices of 1.48 for the lipid phase and 1.33 for water were used in the analysis.

The drug entrapment efficiency of the cubosomes was determined using a centrifugal ultrafiltration approach. To establish the total amount of drug (TMZ or DOX) present in the formulation (*C*_drug_added_), a defined volume of the drug-loaded cubosomal dispersion was dissolved in methanol and analyzed by UV-visible spectroscopy (Cary 60, Agilent, USA), using methanol as the reference. Quantification was performed at *λ* = 329 nm (TMZ) or *λ* = 490 nm (DOX) using a previously constructed calibration curve.

The amount of the drug encapsulated within the cubosomes (*C*_drug_cubosome_) was evaluated by separating free drug from the nanocarriers using Amicon Ultra centrifugal filter units, followed by centrifugation (MPW-352R, MPW MED. INSTRUMENTS, Poland). Encapsulation efficiency was calculated according to the following equation:EE (%) = (*C*_drug_cubosome_/*C*_drug_added_) × 100,where *C*_drug_cubosome_ corresponds to the TMZ or DOX concentration retained within the cubosomes and *C*_drug_added_ denotes the initially introduced drug concentration.

### 2.4 Electrochemical measurements

Electrochemical measurements were performed using a CHI bipotentiostat (CHI750) in a standard three-electrode configuration, with an Ag/AgCl reference electrode, a platinum foil counter electrode, and a glassy carbon working electrode (GCE, surface area of 7.01 mm^2^) modified with a drug-loaded mesophase. Before experiments, the GCE was polished with alumina slurries (0.3 and 0.05 µm), sonicated to remove residual alumina, rinsed with ethanol and water, and dried. Differential pulse voltammetry (DPV) was employed to investigate the drug release from the tested mesophases. The release experiments were designed as a comparative analysis of different cubosome formulations under identical experimental conditions. The electrochemical measurements provide a quantitative signal proportional to the concentration of electroactive drugs in the release medium and were therefore used to compare the relative release behavior of the investigated systems. For release studies, the GCE was modified with a drug-loaded bulk mesophase deposited in a cylindrical Teflon holder, maintaining a constant layer thickness of 0.5 mm. The modified electrode was immediately immersed in the supporting electrolyte, and measurements were performed. Before analysis, the electrolyte was deoxygenated with argon (99.999%) for 15 min, and an argon atmosphere was maintained over the solution during measurements.

### 2.5 Cell culture

HeLa and A172 cell lines were purchased from the American Type Culture Collection (ATCC, USA). Both were grown in RPMI 1640 medium (HyClone, USA) supplemented with 10% (v/v) fetal bovine serum (FBS; HyClone) and Antibiotic Antimycotic Solution (Sigma-Aldrich). All cells were incubated at 37 °C in a humidified atmosphere containing 5% CO_2_.

### 2.6 Cell viability (MTS-based assay)

The viability of the tested cells was assessed using an MTS-based assay (CellTiter 96 AQueous One Solution Cell Proliferation MTS Assay; Promega, Germany), as previously described^23^ with minor modifications. Cells were seeded in 96-well plates at a density of 4 × 10^3^ cells per well in 100 µL of complete growth medium and incubated overnight. The following day, the medium was supplemented with either DOX- or TMZ-loaded MO or MP nanoparticles (drug concentrations of 1.25 µM or 100 µM, respectively). Untreated cells served as controls. After 24 h of treatment, 20 µL of the MTS reagent was added to each well, and the plates were incubated for an additional 3 h. Absorbance was measured at 490 nm using a Synergy2 microplate reader (BioTek Instruments, USA). Cell viability was expressed as a percentage relative to control cells, which were set at 100%.

### 2.7 Trypan blue-based dye exclusion assay

The viability of the treated cells (A172 and T98G) was evaluated using the trypan blue-based assay, as previously described,^23^ with minor changes. Briefly, 1 × 10^5^ cells were seeded in each well of a 12-well plate in 1 mL of complete growth medium. After 24 h, the medium was supplemented with DOX-, TMZ-, or DOX/TMZ-loaded MO or MP nanoparticles (drug concentrations of 1.25 µM or 100 µM, respectively). Cells not exposed to the drug or nanoparticles served as controls. After 24 h of incubation, all the cells were harvested, pelleted by centrifugation (200 × *g* for 5 min), resuspended in Dulbecco's phosphate-buffered saline (D-PBS; Sigma-Aldrich), and stained with trypan blue (NanoEnTek, South Korea) at a final concentration of 0.2% (w/v) for 10 min. The number of viable and necrotic cells was determined using an EVE Automatic Cell Counter (NanoEnTek), and the results were expressed as a percentage of viable cells compared to the non-treated controls.

### 2.8 Confocal microscopy

Cells (1 × 10^5^) were seeded onto glass coverslips and incubated for 24 h. The following day, the cells were treated with designated concentrations of DOX-, TMZ-, or DOX/TMZ-loaded MO or MP nanoparticles (1.25 µM, DOX; 100 µM, TMZ). After 18 h of incubation, the coverslips were washed with PBS, and the cells were fixed with 4% (w/v) paraformaldehyde (Thermo Scientific) for 10 min. The fixed cells were permeabilized with 0.25% (v/v) Triton X-100 in PBS (Sigma-Aldrich) for 10 min and blocked with 3% (w/v) bovine serum albumin (BSA; Sigma-Aldrich) in PBS for 45 min. The structure of the cells was visualized with phalloidin-FITC (2 µg mL^−1^ in PBS; Sigma-Aldrich) for 30 min. Nuclei were counterstained with DAPI (4′,6-diamino-2-phenylindole dihydrochloride; 1 µg mL^−1^; Sigma-Aldrich) in deionized water for 2 min. Washed coverslips were mounted on regular microscope slides using fluorescence mounting medium (Dako, USA). The confocal images were acquired using a Zeiss LSM800 microscope equipped with a plan-apochromatic 63×/1.4 oil DIC M27 objective lens. The fluorescence from Alexa Fluor 647, DOX, and DAPI was measured at *λ*_ex_ 650/*λ*_em_ 670 nm, *λ*_ex_ 470/*λ*_em_ 555 nm, and *λ*_ex_ 340/*λ*_em_ 488 nm, respectively. The fluorescence signal from phalloidin-FITC was detected at *λ*_ex_ 495/*λ*_em_ 520 nm. Images were processed using ZEN 2.1 software (Zeiss, Germany) and exported in TIFF format.

### 2.9 Data analysis

All experiments were performed in at least triplicate. Quantitative data are expressed as the mean ± standard deviation (SD). Data were analyzed using GraphPad Prism version 10.6.1 for Windows (GraphPad Software, Inc., USA). Statistical analyses were performed using the Mann–Whitney *U* test or one-way ANOVA followed by Bonferroni's *post hoc* test. Statistical significance was defined as *p* < 0.05.

The synergistic effect of DOX and TMZ loaded into MO or MP cubosomes was evaluated using the Bliss independence method:^27^*R*_C_ = *R*_1_ + *R*_2_ − (*R*_1_ × *R*_2_), where *R*_C_ is the expected response of the combination and *R*_1_ and *R*_2_ are the responses of the individual drugs.

## 3 Results and discussion

### 3.1 Phase behavior and characterization of dual drug-loaded mesophases

Small-angle X-ray scattering was employed to determine the structural parameters of cubic phases containing two incorporated drugs. To evaluate the effect of dual-drug loading, the samples containing only a single drug (either DOX or TMZ) as well as drug-free cubic phases were also analyzed. Both MP and MO were used to prepare fully hydrated TMZ-loaded cubic phases. Due to its shorter hydrophobic tail, MP forms cubic phases with larger aqueous channels compared to those of MO. The enlarged channel size in the MP-based cubic phase facilitated the release of drug molecules, as we recently demonstrated.^23^

Cubic phases prepared from MO and MP at pH 5.3 exhibited *Pn*3̄*m* diamond symmetry, indicated by six characteristic peaks with *q* ratios of √2, √3, √4, √6, √8, and √9 (Fig. 1A and B). Analysis of the diffractograms revealed that neither the addition of a single drug (DOX or TMZ) nor the simultaneous incorporation of both drugs affected the cubic phase symmetry. Furthermore, increasing the temperature to 37 °C did not alter the *Pn*3̄*m* diamond symmetry in either the MO : buffer or MP : buffer systems, as confirmed by the SAXS patterns.

**Fig. 1:**
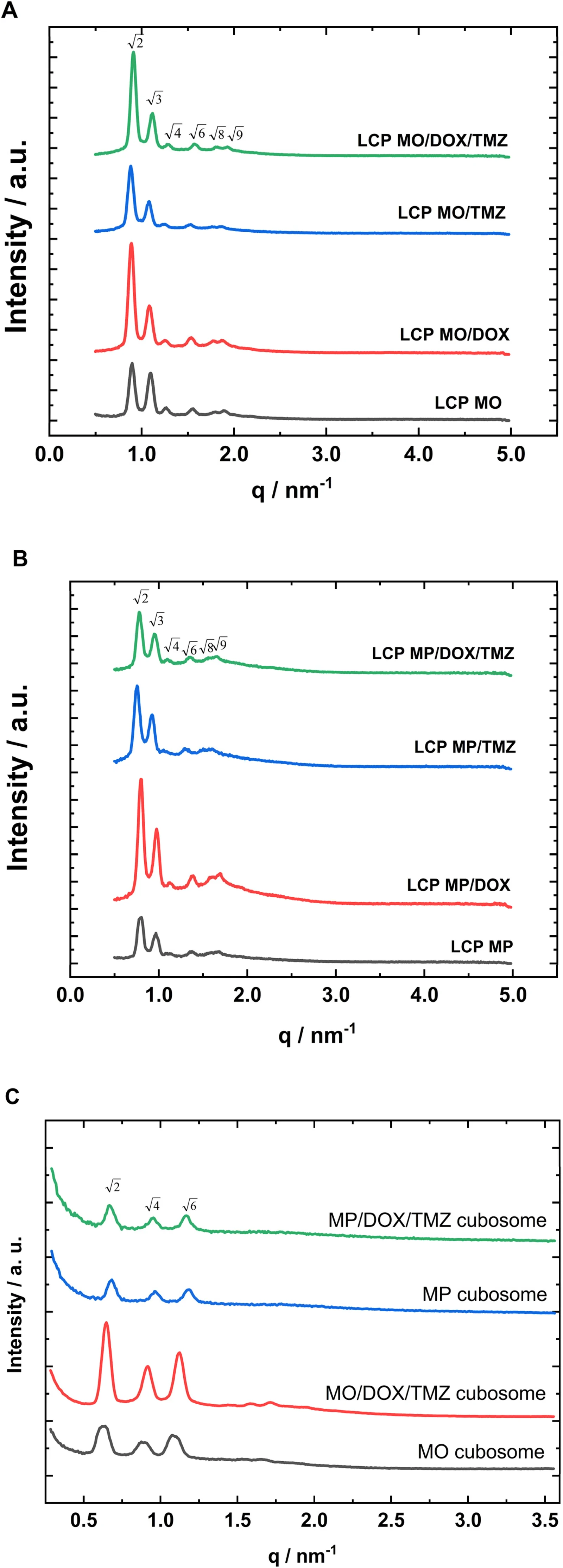
(A) Diffractograms of cubic phases prepared from monoolein (MO) and acetate buffer, doped with doxorubicin (DOX) and temozolomide (TMZ) at a 3 : 1 molar ratio; (B) diffractograms of cubic phases prepared from monopalmitolein (MP) and doped with doxorubicin and temozolomide at a 3 : 1 molar ratio; (C) diffractograms of cubosomes prepared from monoolein or monopalmitolein co-loaded with doxorubicin and temozolomide at a 1 : 80 molar ratio.

The structural parameters of the cubosomes were calculated based on the Infinite Periodic Minimal Surface (IPMS) model.^28–30^ The lipid length (*l*) and water channel diameter (*d*) were calculated using the equations provided in the SI (S1).

Analysis of the structural parameters (Table 1) indicated that neither temperature nor drug loading affects the lattice parameter (*a*) of MO and MP cubic phases. Incorporation of doxorubicin slightly decreases the lattice parameter, whereas TMZ caused an increase, likely due to its localization at the lipid–water interface, which flattens the lipid monolayer and expands the aqueous channels (*d*). In MP-based phases, DOX had little effect on the channel diameter, while in MO-based phases, it slightly reduced it. Simultaneous loading of DOX and TMZ results in a modest increase in the lattice parameter, though values remained below those of TMZ-only phases. Dual-drug loading did not significantly alter the channel size in MP phases but slightly enlarged it in the MO phases. Increasing the temperature to 37 °C consistently reduced both the lattice parameter and the aqueous channel diameter across all formulations, reflecting decreased hydration of the polar head-groups and enhanced negative curvature. Additionally, the type of lipid influenced channel dimensions: MP-based cubic phases exhibit aqueous channels approximately 1.2 nm wider than those of MO-based phases.

**Table 1:** Parameters for temozolomide (TMZ)- and doxorubicin (DOX)-loaded monoolein (MO)- and monopalmitolein (MP)-derived liquid-crystalline cubic phases (LCPs)

Formulation	Symmetry	Lattice parameter *a* [nm], 24 °C	Lattice parameter *a* [nm], 37 °C	Water channel diameter *d* [nm], 24 °C	Water channel diameter *d* [nm], 37 °C
LCP MO	*Pn*3̄*m*	9.9	9.2	4.3	3.8
LCP MO/DOX	*Pn*3̄*m*	9.6	9.2	4.1	3.8
LCP MO/TMZ	*Pn*3̄*m*	10.7	10.1	5.0	4.5
LCP MO/DOX/TMZ	*Pn*3̄*m*	10.3	9.8	4.7	4.3
LCP MP	*Pn*3̄*m*	11.2	10.7	5.8	5.4
LCP MP/DOX	*Pn*3̄*m*	11.1	10.6	5.7	5.5
LCP MP/TMZ	*Pn*3̄*m*	11.8	11.4	6.2	5.9
LCP MP/DOX/TMZ	*Pn*3̄*m*	11.4	11.0	5.9	5.6

The MO and MP cubosomes displayed three peaks, with ratios of √2 : √4 : √6, indicating *Im*3̄*m* symmetry (Fig. 1C). The crystal lattice parameter and aqueous channel diameter were determined for the cubosomes. The calculated structural parameters are summarized in Table 2. For MP-based cubosomes, the water channel width was observed to be slightly larger compared to that of MO-based systems.

**Table 2:** Phase and lattice parameters measured using small-angle X-ray scattering. The size distribution and polydispersity index (PDI) were obtained using dynamic light scattering. DOX, doxorubicin; EE%, encapsulation efficiency (%); MO, monoolein; MP, monopalmitolein; TMZ, temozolomide

Cubosome formulation	Symmetry	*a* [nm]	*d* [nm]	Hydrodynamic size [nm]	PDI	EE%
MO	*Im*3̄*m*	14.1	5.2	180.6 ± 1.9	0.230 ± 0.01	—
MO/DOX/TMZ	*Im*3̄*m*	14.4	5.4	174.1 ± 0.9	0.294 ± 0.04	99 ± 1.7 (DOX)
						89 ± 3.7 (TMZ)
MP	*Im*3̄*m*	12.8	4.8	166.4 ± 3.0	0.231 ± 0.07	—
MP/DOX/TMZ	*Im*3̄*m*	12.8	4.8	178.9 ± 3.4	0.259 ± 0.07	99 ± 0.7 (DOX)
						87 ± 6.9 (TMZ)

DLS was employed to characterize the physicochemical properties of the cubosomes. The particle size distributions and polydispersity index (PDI) values are summarized in Table 2, which shows that all formulations exhibited sizes below 200 nm and PDI values below 0.3.

The size of the cubosomal formulations increased over one week of storage from 174.1 ± 0.9 to 233.0 ± 14.0 nm for MO and from 178.9 ± 3.4 to 257.3 ± 20.3 nm for MP. In addition, the PDI has increased slightly from 0.294 ± 0.04 to 0.37 ± 0.05 and from 0.27 ± 0.07 to 0.38 ± 0.04, respectively, indicating a slight decrease in colloidal stability during storage (Table S1).

### 3.2 Electrochemical characterization of drugs incorporated into lipid mesophases – *in vitro* release study

To evaluate the separation of the reduction peaks, differential pulse voltammetry experiments were conducted in a MES solution (pH 5.3), where both DOX and TMZ were simultaneously dissolved (Fig. 2A). The resulting voltammograms were analyzed to study the electrochemical behavior of both substances. Two distinct reduction peaks were recorded: one corresponding to DOX, with a peak potential at *E*_DOX_ = −0.50 V, associated with the reduction of the quinone group of DOX, and another corresponding to TMZ, with a peak potential at *E*_TMZ_ = −0.84 V, related to the reduction of the tetrazinone ring. These findings confirm that both drugs can be simultaneously monitored with high accuracy using electrochemical methods, providing a reliable basis for further studies on their release profiles and therapeutic efficacy.

**Fig. 2:**
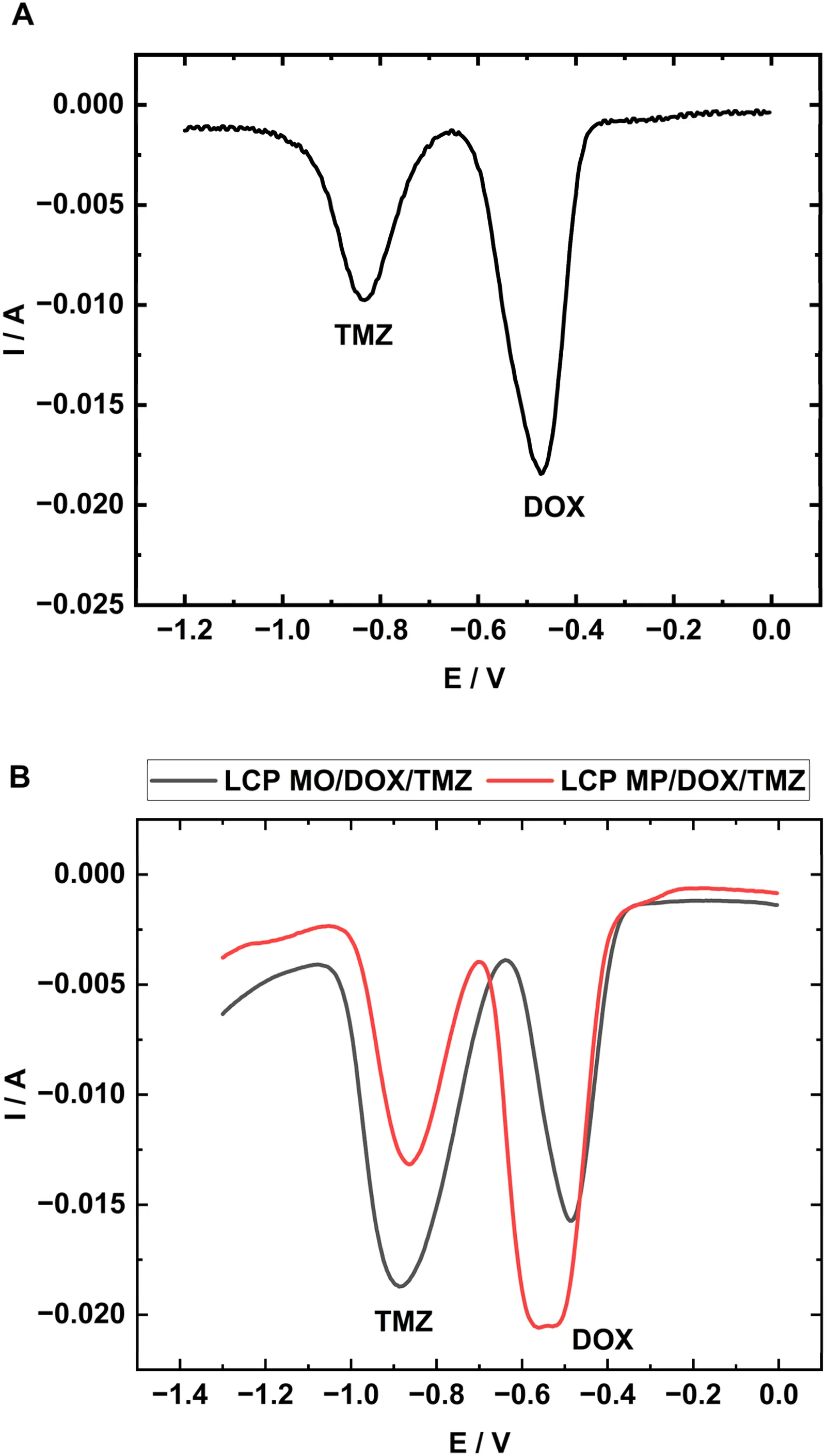
(A) Differential pulse voltammogram obtained at a glassy carbon electrode (GCE) in MES buffer (pH 5.3) with a doxorubicin : temozolomide (DOX : TMZ) molar ratio of 1 : 1; (B) differential pulse voltammograms recorded at a GCE modified with monoolein (MO)- and monopalmitolein (MP)-based liquid-crystalline cubic phases (LCPs), doped with doxorubicin and temozolomide at a 3 : 1 molar ratio.

The DPV results indicate that encapsulation of the anticancer drugs in MO and MP cubic phases caused minor shifts in the reduction potentials of DOX and TMZ (Fig. 2B). For the tetrazinone ring reduction in MO and MP cubic phases, the potential shifted approximately 0.05 V toward more negative values compared to that of the reduction peaks observed in the DPV curves of the drug solutions. Immobilization of DOX in MP cubosomes shifted the reduction potential from *E* = −0.47 V in solution to *E* = −0.54 V within the cubic phase. Similarly, DOX encapsulated in MO cubosomes exhibited a 0.06 V shift toward more negative potentials relative to its reduction peak in solution.

The effect of co-immobilizing two drugs on the release rates of DOX and TMZ from the cubic phases was also investigated. The drug release was examined under conditions where either a single drug or both drugs were immobilized in the cubic phase (Fig. 3). The release profiles show that incorporating TMZ into the cubic phase reduced the release rate of DOX in the MO cubic phase. Fig. S1 presents the normalized release rates of DOX and TMZ from MO- and MP-derived cubic phases loaded with both drugs. After 60 minutes, DOX release from the cubic phase with both drugs was 25% lower compared to that of the phase containing only DOX. However, the release rate of DOX from the MP phase was similar in both single- and dual-drug systems. Table S2 summarizes the release kinetics model fitting parameters obtained for all investigated systems. The applied kinetic models are described in ref. 23. In MO cubic phases, the Korsmeyer–Peppas model indicated diffusion-controlled drug release, with *n* values close to 0.5. In contrast, in the phases containing both drugs, *n* values of 0.6 suggested anomalous (non-Fickian) transport, indicating the presence of drug–carrier interactions. This behavior was also reflected in the release profiles, where a reduced release of DOX from the MO cubic phase was observed. *n* values higher than 0.6 observed for TMZ release, regardless of the formulation tested, indicate that the process is not purely diffusion-controlled but follows an anomalous (non-Fickian) transport mechanism. Adding DOX to the cubic phase enhanced TMZ release. After 60 min, 70% of TMZ was released from the dual-drug phase, compared to 60% from the TMZ-only phase. Similarly, in the MP phase, 95% of TMZ was released from the dual-drug system *vs.* 85% from the phase containing only TMZ. The observed decrease in TMZ current may include also partial TMZ hydrolysis, likely driven by local pH shifts or electrostatic interactions with positively charged DOX. The electroactive products of the chemical degradation of TMZ can be evaluated electrochemically by monitoring their electrooxidation currents at positive potentials. Voltammetric analysis demonstrated that maintaining a high TMZ : DOX ratio is essential to preserve drug stability and ensure therapeutic synergy, regardless of the lipid phase geometry.

**Fig. 3:**
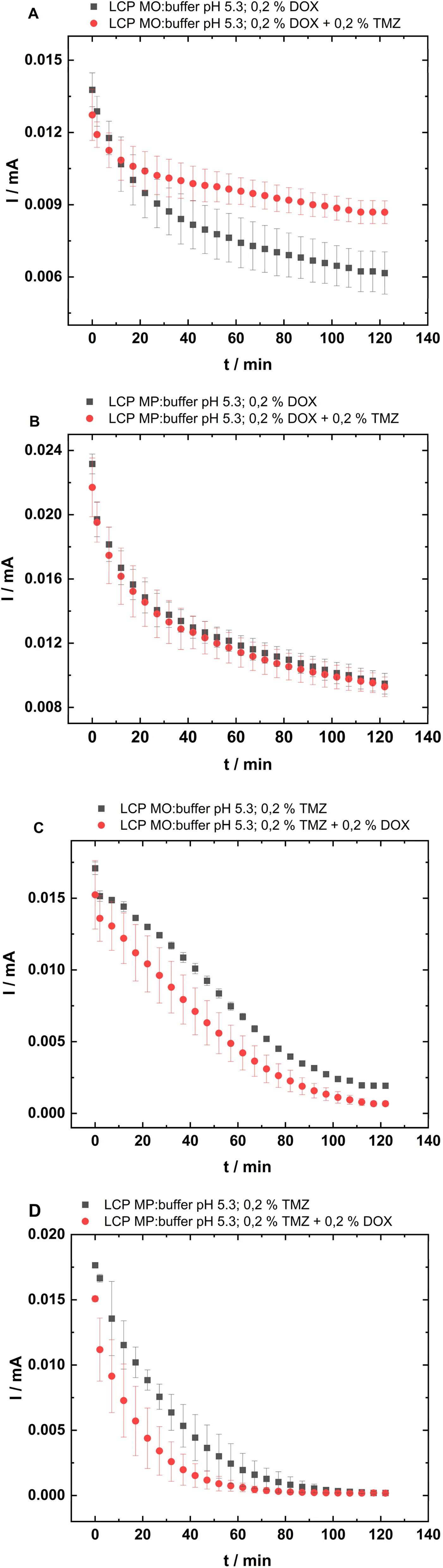
Drug release profiles for monoolein (MO) (A and C) and monopalmitolein (MP) (B and D) liquid-crystalline cubic phases (LCPs) in single- and dual-drug systems: doxorubicin (DOX) (A and B) and temozolomide (TMZ) (C and D).

DPV measurements were also performed under conditions simulating cell-based studies, using a higher TMZ concentration and a reduced DOX concentration (DOX : TMZ molar ratio of 1 : 80) (Fig. 4). Temozolomide was applied at higher molar concentrations than doxorubicin due to its substantially lower cytotoxic potency reported in the literature. While TMZ typically exhibits biological activity in the high micromolar range, DOX remains effective at nano- to low micromolar concentrations. Similar TMZ/DOX concentration ranges have previously been reported in glioblastoma studies.^19,22^ The reduction peaks for DOX and TMZ remained well-separated, enabling accurate determination of the release profile, which is essential for understanding drug release kinetics and their therapeutic potential in cellular environments.

**Fig. 4:**
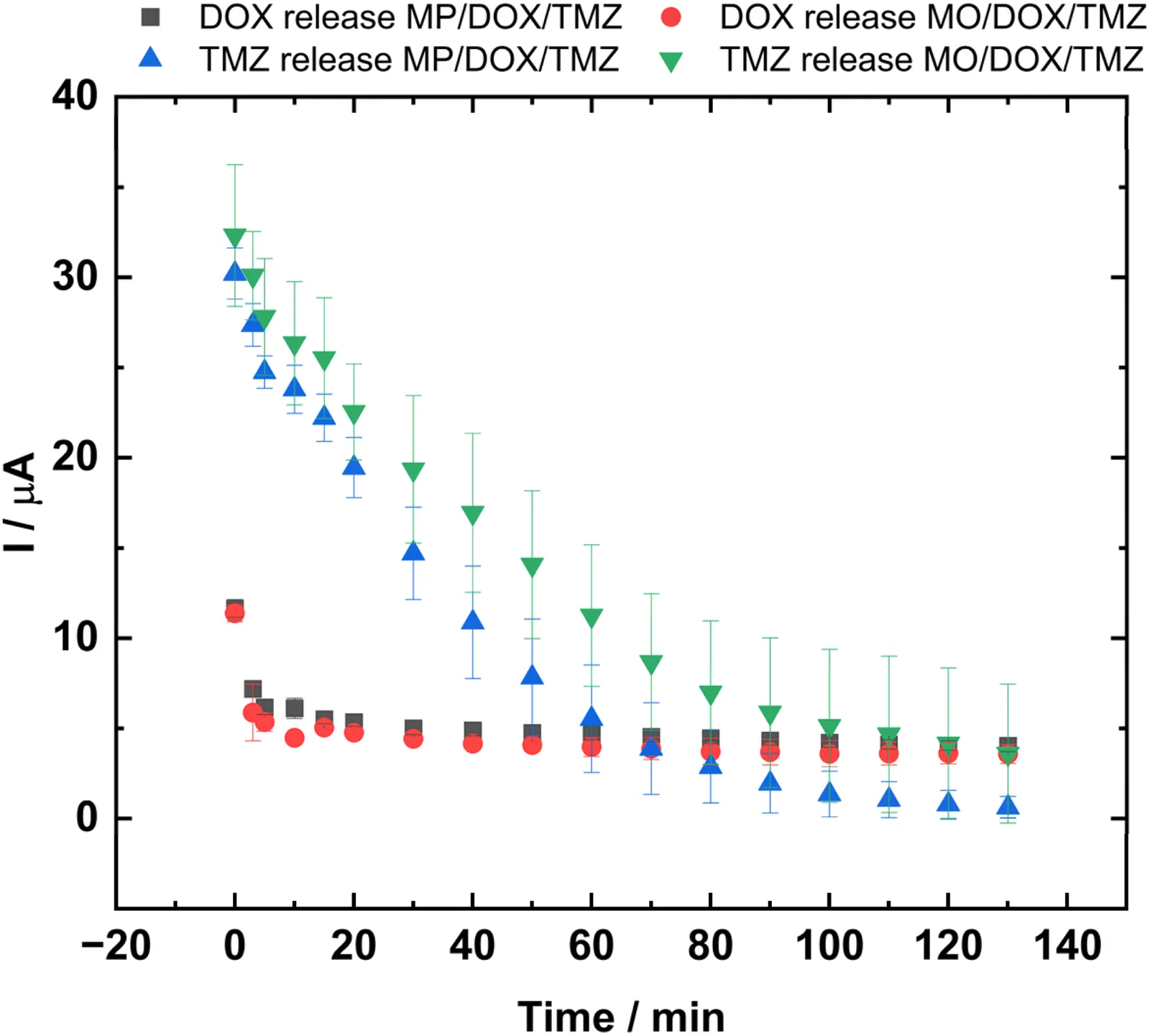
Drug release from monoolein (MO) or monopalmitolein (MP) liquid-crystalline cubic phases (LCPs) loaded with doxorubicin (DOX) and temozolomide (TMZ) at a 1 : 80 molar ratio.

In carriers containing both DOX and TMZ at a molar ratio of 1 : 80, changing the lipid did not significantly affect the release rate of DOX. However, a slight increase in the release rate of TMZ was observed when the lipid was changed to MP. This change in the release rate is likely due to differences in the water-channel structure of the lipid phases, which may facilitate more efficient release of TMZ in the MP phase compared to that in the MO-based cubic phase.

### 3.3 Analysis of the biological properties of dual drug-loaded mesophases

In previous studies, we reported that TMZ-loaded MPs exhibited greater toxicity than TMZ-loaded MO phases.^23^ Here, we further explored the anticancer properties of the MO and MP phases through a side-by-side comparison of DOX- and TMZ-co-loaded (DOX/TMZ) particles. Two cell lines of different origins were used: A172 (GBM-derived) and HeLa (cervical cancer-derived). The analysis was performed using two classical assays: MTS and trypan blue staining (Fig. 5). Fig. S2 shows the dose–response relationship between cubosome concentration and cell viability in cells exposed to empty cubosomes. It was confirmed that 100 µM TMZ loaded into both MO and MP particles does not significantly affect cell survival. In the case of phases loaded with 1.25 µM DOX, a limited toxic effect was observed, especially for MP/DOX cubosomes, which is consistent with our previous observations.^23^ Both cell lines treated with the DOX/TMZ combination loaded into MO or MP phases exhibited the most potent anticancer activity. This synergistic effect of co-administered TMZ and DOX was confirmed using the Bliss independence method.^27^ Notably, the MP-based formulation (MP/DOX/TMZ) showed the most pronounced efficacy. This supports our previous data indicating that drug-loaded MP cubosomes are most effective at the tested concentrations.^23^ Overall, both MP and MO formulations can be considered potential multi-drug carriers as they significantly alter cancer cell survival rates. This observation was further supported by confocal imaging of the tested cells. As presented in Fig. 6, MP-based cubosomes exhibited the most potent anticancer activity. Cells treated with MP carriers co-loaded with DOX and TMZ showed the most severe structural damage, with DOX accumulation reaching its highest levels among all tested groups. These findings align with our previous data^23^ and suggest that while both MP and MO formulations are viable multi-drug carriers, the MP-based systems offer improved efficacy. This superior performance of MP drug-loaded cubosomes is likely due to their unique structure and higher loading capacity of drugs, which ultimately result in a significant reduction in cancer cell viability.

**Fig. 5:**
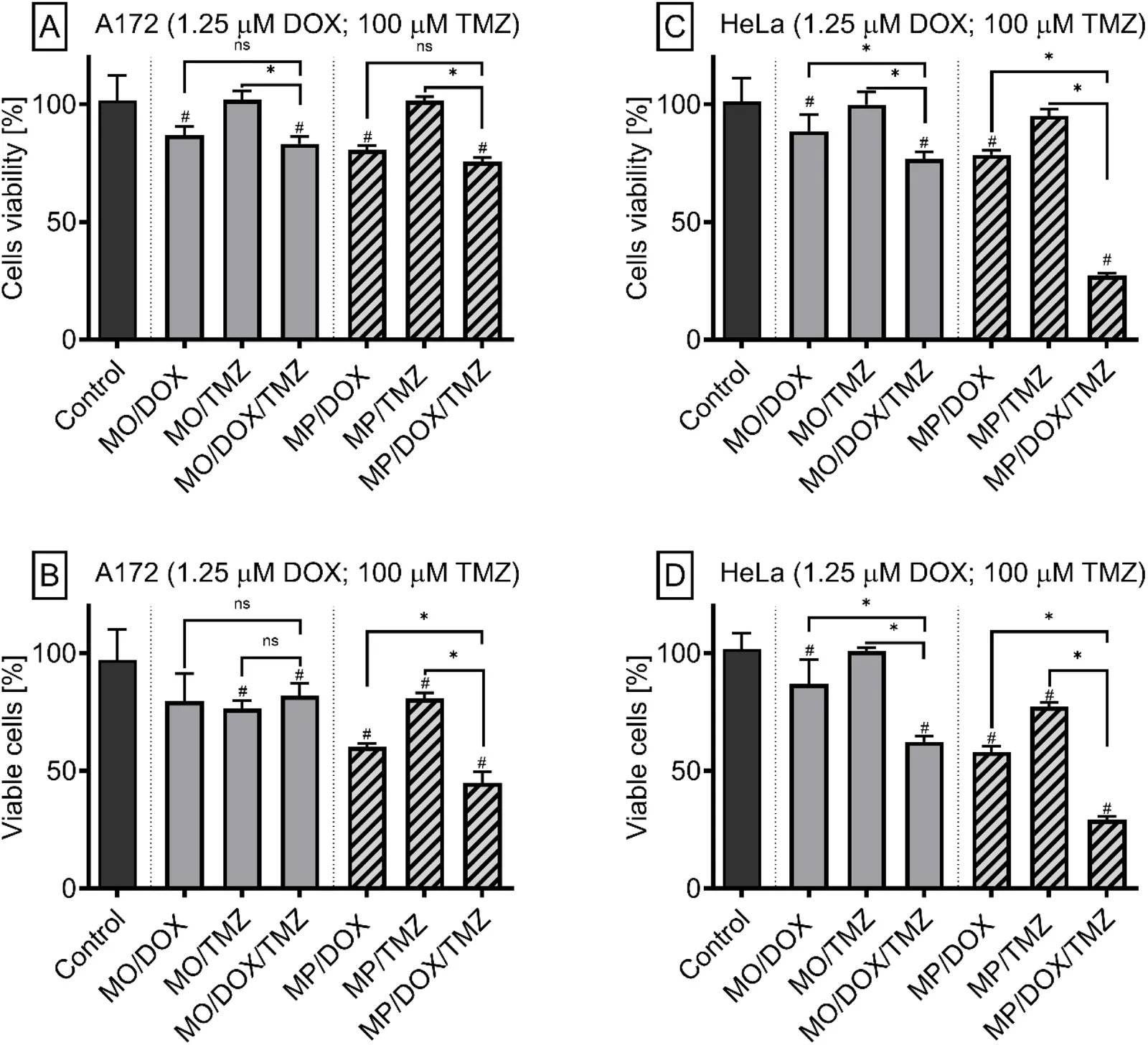
Analysis of the cell viability of A172 and HeLa cells treated with doxorubicin (DOX)- or TMZ- (TMZ) or DOX/TMZ-loaded MO or MP phases (MO/TMZ; MO/DOX; MO/DOX/TMZ or MP/DOX, MP/TMZ and MP/DOX/TMZ, respectively). The number of viable cells was assessed using MTS (A and C) and trypan blue exclusion (B and D) assays. Data are presented as mean ± SD (standard deviation); (*n* > 10). Non-treated cells served as controls. * Represents statistically significant effect (*p* < 0.05) between designated experimental groups. ^#^ Represents statistically significant effect (*p* < 0.05) between the designated experimental group and the control.

**Fig. 6:**
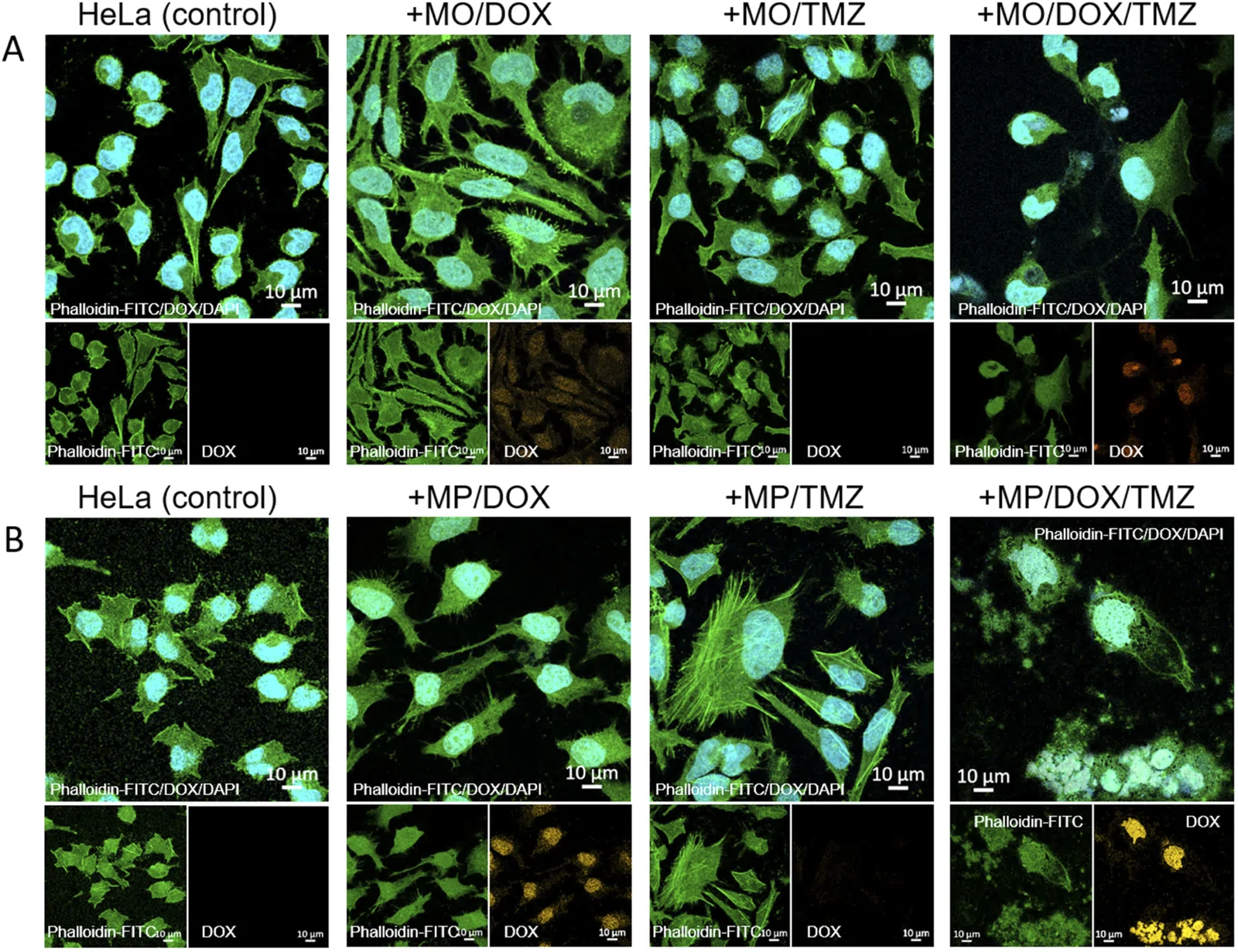
Representative images of HeLa cells treated with (A) monoolein (MO) and (B) monopalmitolein (MP) phases containing doxorubicin (DOX), temozolomide (TMZ), or a DOX/TMZ combination. Cell architecture is visualized in green (FITC-conjugated phalloidin), while nuclei are counterstained in blue (DAPI). Red fluorescence indicates the intracellular accumulation of DOX. All images were captured using a 63×/1.4 oil DIC M27 objective.

Because a major obstacle in brain tumor therapy is the limited penetration of therapeutic agents across the BBB, cubosomes, due to their nature, exhibit an affinity for cell membranes and may therefore promote interactions with endothelial cell membranes.^15^ Previous studies have suggested that lipid nanocarriers are able to cross the BBB through mechanisms such as adsorptive-mediated transcytosis, endocytosis-related transport, or membrane fusion processes.^31,32^ Nevertheless, future studies using established *in vitro* models (*e.g.*, PAMPA, MDCK-II, or hCMEC/D3 systems) are necessary to determine whether dual-loaded cubosomes (MO and MP) can effectively cross the BBB.^33–35^ In particular, functionalizing cubosomes with targeting ligands and employing alternative routes of administration, such as the intranasal route, are highly worth considering in the near future.^36–38^

## 4 Conclusions

This study demonstrates the utility of voltammetric monitoring for drug release from lipidic nanocarriers, which are increasingly employed to deliver cytotoxic agents in cancer therapies. Here, we show that this approach is effective for the simultaneous immobilization of DOX and TMZ within the cubic phase and cubosomes, which is advantageous from the therapeutic point of view. The electrochemical methods enable the determination of the content of each drug in the carrier mesophase and evaluation of the contribution of its decomposition processes, expanding upon previous reports that the degradation pathway can be tracked electrochemically.^18,23,25^ In the case of our dual-drug cubic phase carrier, the separation of the differential-pulse voltammetry peaks for both drugs enables precise monitoring of their release profiles and evaluation of the release mechanism. The differences in the ratios of the voltammetric signals over time and the mesophase used may help explain the drug's possible interactions, especially the influence of interactions with the lipid component forming the drug delivery vehicle. These physicochemical observations could be pivotal for the development of advanced anticancer drug delivery systems.

## Author contributions

ED: investigation, data curation; DG: conceptualization; investigation, formal analysis, methodology, data curation, writing – original draft; MG: investigation, formal analysis, methodology, data curation; EG: investigation, formal analysis, methodology, data curation; RB: methodology, supervision, validation, writing – review and editing; EN: conceptualization, methodology, supervision, data curation, validation, writing – original draft, writing – review and editing.

## Conflicts of interest

The authors declare no competing interests.

## Supplementary Material

NA-008-D6NA00459H-s001

## Data Availability

Raw data in alternative formats are available from the corresponding author upon reasonable request. All data supporting the findings of this study are included in the paper and its supplementary information (SI). Supplementary information is available. See DOI: https://doi.org/10.1039/d6na00459h.
