# Supplementary material for: Dual-drug delivery system based on cubosomes for glioblastoma therapy: electrochemical, structural and biological characterization
Source: Nanoscale Adv. 2026 Aug 18;8(20):5943–53. doi: 10.1039/d6na00459h (PMC13559538; doi:10.1039/d6na00459h)
Supplement: Supplementary file 1 [file NA-008-D6NA00459H-s001.pdf]

Supplementary Information (SI) for Nanoscale Advances.
This journal is © The Royal Society of Chemistry 2026

Dual-drug delivery system based on cubosomes for glioblastoma therapy: electrochemical, structural, and biological properties

Ewelina Dąbrowska[1], Ewa Gajda[2], Marlena Godlewska, Damian Gaweł[2], Renata Bilewicz[1], Ewa Nazaruk[1*]

[1]Faculty of Chemistry, University of Warsaw, Pasteura 1, Warsaw 02-093, Poland

[2]Department of Cell Biology and Immunology, Centre of Postgraduate Medical Education, Marymoncka 99/103, Warsaw 01-813, Poland

Supporting Information

Scheme 1. Chemical structures of the used reagents. A) Monoolein (1-oleoyl-rac-glycerol; MO); B) Monopalmitolein (1-(9Z-hexadecenoyl)-rac-glycerol; MP); C) Pluronic F127; D) Chemical structure and redox reaction of temozolomide (TMZ) E) Chemical structure and redox reaction of doxorubicin (DOX).

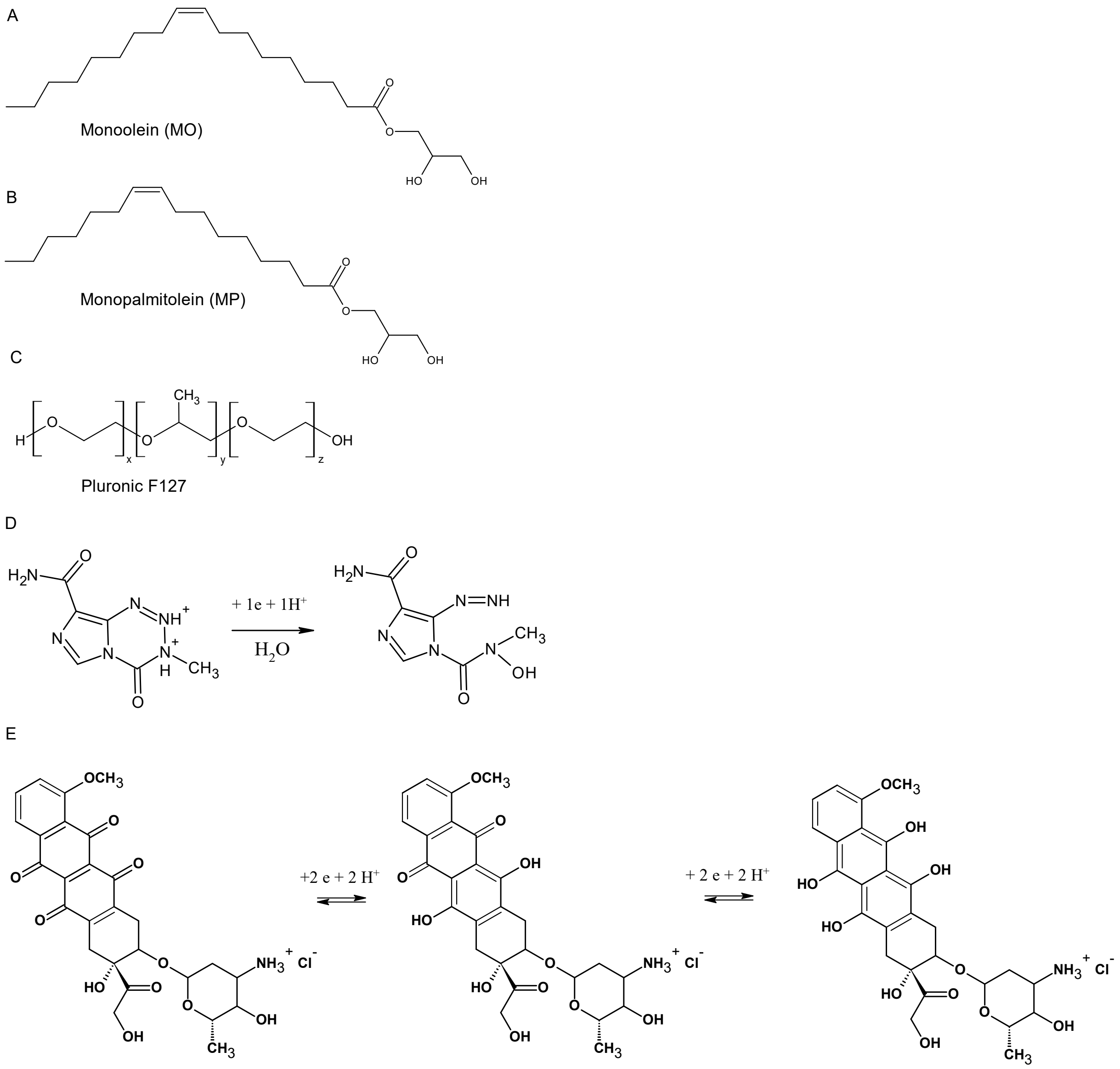

S1. The structural parameters characterizing the cubosomes were calculated based on the Infinite Periodic Minimal Surface (IPMS) model. [1] [2] [3] In order to calculate the lipid length (l) and water channel diameter (d), the following calculations were performed:

a) lattice parameter (a)

$$a = \frac{2\pi\sqrt{h^2 + k^2 + l^2}}{q_0}$$

where: q0 – value for the first peak in the diffraction pattern; hkl – Miller indices for the first peak in the diffraction pattern;

b) weight fraction of water (φ_w)

$$\varphi_w = \frac{m_w}{m_w + (1 - m_w)\frac{d_w}{d_l}}$$

where: mw – weight of the water fraction, dw – density of the water phase (0.997 g/cm3), dl – lipid density (0.942 g/cm3 for monoolein or 0.982 g/cm3 for monopalmitolein)

c) weight fraction of the lipid (φ_l)

$$\varphi_l = 1 - \varphi_w$$

d) lipid length (l)

$$\varphi_l = 2\sigma\left(\frac{l}{a}\right) + \frac{4}{3}\pi\chi\left(\frac{l}{a}\right)^3$$

where: σ – constant value of 1.919 for the Pn3m phase; χ – constant value of -2 for the Pn3m phase

e) the radius of the water channel (r_w)

$$r_w = \left(-\frac{\sigma}{2\pi\chi}\right)^{1/2} a - l$$

Table S1 The physical stability of MO- and MP-derived cubosomes loaded with DOX and TMZ.

SAMPLE	Size (nm)	PDI
**MO/DOX/TMZ freshly prepared**	174.1 ± 0,9	0,294 ± 0.04
**MO/DOX/TMZ after 24h**	177.6 ± 2.0	0.30 ± 0.07
**MO/DOX/TMZ after 48h**	189.3 ± 12.8	0.31 ± 0.02
**MO/DOX/TMZ after 72h**	202.7 ± 15.3	0.33± 0.02
**MO/DOX/TMZ after week**	233.0± 14.0	0.37 ± 0.05
**MP/DOX/TMZ freshly prepared**	178.9 ± 3.4	0.27 ± 0.07
**MP/DOX/TMZ after 24h**	183.9± 5.0	0.27 ± 0.05
**MP/DOX/TMZ after 48h**	203.6 ± 9.0	0.30± 0.05
**MP/DOX/TMZ after 72h**	216.7 ± 15.5	0.32 ± 0.03

**MP/DOX/TMZ after week**	257.3 ± 20.3	0.38 ± 0.04

S2. Kinetic models

The drug release data were fitted to the Higuchi model: $M_t / M_\infty = k\sqrt{t}$, where M_t/M_∞ is the fraction of drug released at time *t*, and *k* is the release rate constant. In this model, the cumulative drug release is proportional to the square root of time. The Korsmeyer–Peppas model, $M_t / M_\infty = k_1\, t^n$, was alsko applied. In this model *k1* is a kinetic constant, *t* is the release time and *n* is the diffusional exponent. The diffusional exponent indicates the drug release mechanism. For n = 0.5 the drug release mechanism is Fickian diffusion, while for n = 1, Case II transport occurs. When the "n" value is between 0.5 and 1, it indicates anomalous transport.

Table S2 The release kinetics model fitting parameters

LCP formulation (monitoring DOX concentration)	I order model	Higuchi model	Korsmeyer-Peppas model	
	R^2	R^2	R^2	n
**LCP MO/DOX**	0,970	0,992	0,873	0,55
**LCP MO/DOX/TMZ**	0,959	0,961	0,914	0,60
**LCP MP/DOX**	0,969	0,984	0,996	0.50
**LCP MP/DOX/TMZ**	0,932	0,980	0,978	0,48
**LCP formulation (monitoring TMZ concentration))**	**I order model**	**Higuchi model**	**Korsmeyer-Peppas model**	
	R^2	R^2	R^2	n
**LCP MO/TMZ**	0,917	0,837	0,995	0,65
**LCP MO/DOX/TMZ**	0,963	0,907	0,980	0,77
**LCP MP/TMZ**	0,983	0,955	0,992	0,72
**LCP MP/ DOX/TMZ**	0,997	0,985	0,999	0,55

S3 Synergy calculation

Group	mean_MTS1	mean_MTS2	mean_MTS3	value_1	value_2	value_3	mean
Control	97.84	105.46	96.70	0.0216	-0.0546	0.0330	0.0000
MO/DOX	88.48	86.75	85.07	0.1152	0.1325	0.1493	0.1323
MO/TMZ	104.15	102.08	103.37	-0.0415	-0.0208	-0.0337	-0.0320
MO/DOX/TMZ	80.58	83.26	87.06	0.1942	0.1674	0.1294	0.1636
MP/DOX	82.09	80.72	81.46	0.1791	0.1928	0.1854	0.1858
MP/TMZ	103.54	101.66	101.94	-0.0354	-0.0166	-0.0194	-0.0238
MP/DOX/TMZ	74.67	75.62	74.42	0.2533	0.2438	0.2558	0.2510

Bliss independence

$R_C = R_1 + R_2 - (R_1 \times R_2)$

R_C - expected response of the combinationR1 and R2 are the responses of the individual drugs.
R1; R2 - responses of the individual drugs

MO/DOX/TMZ

R_C=0,1323+(-0,0320)-(0,1323x(-0,0320))
R_C= 0.104571576

MO/DOX/TMZ	R_C
0.1636	0.1046

0,1636>0,1046 →Synergy

MP/DOX/TMZ

R_C=0,1885+(-0,0238)-(0,1885x(-0,0238))
R_C= 0.166371122

MO/DOX/TMZ	R_C
0.2510	0.1664

0,2510>0,1664 →Synergy

Fig. S1 Normalized release rates of DOX and TMZ from MO- and MP-derived cubic phases loaded with both drugs.

A)

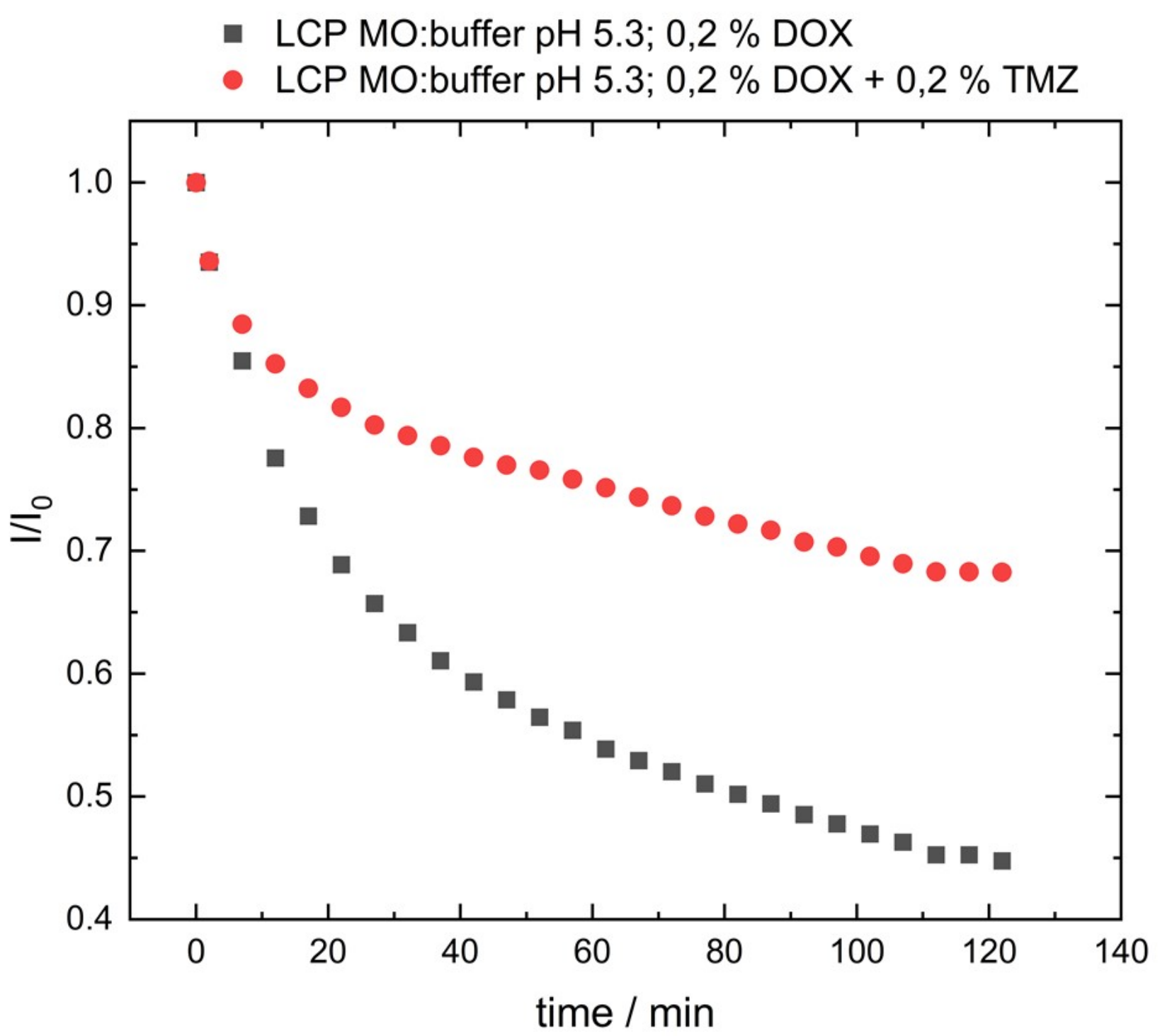

B)

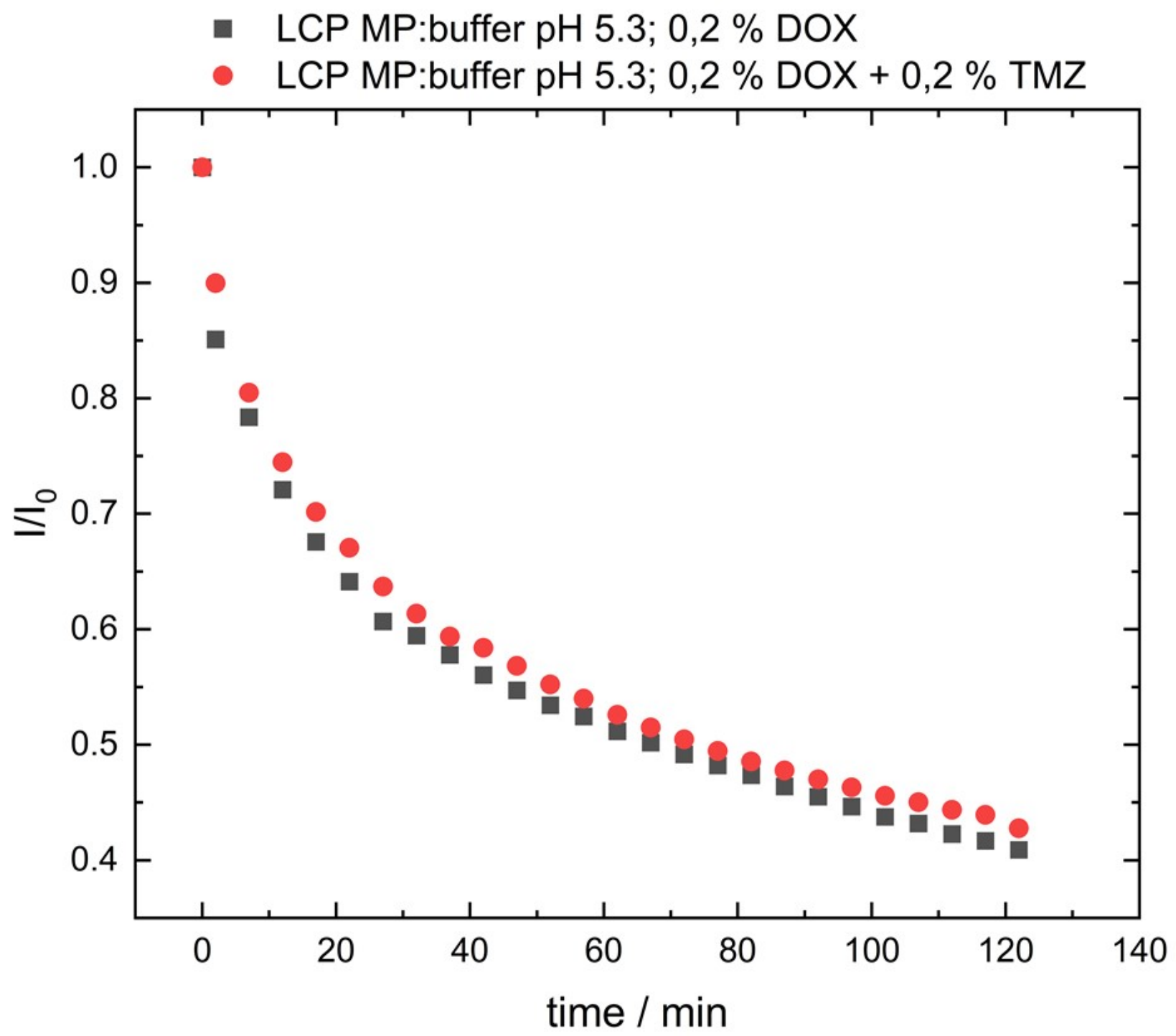

C)

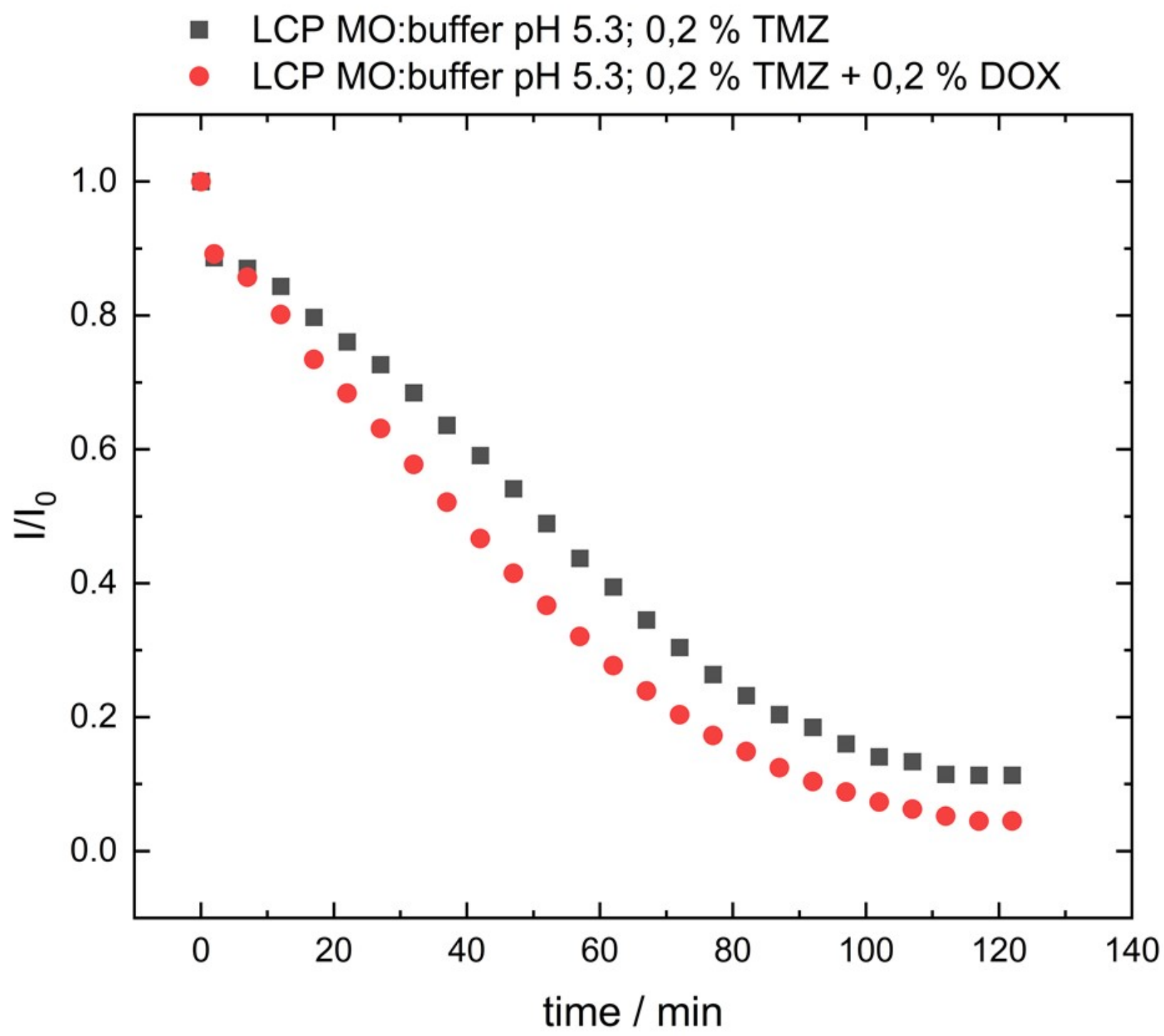
LCP MO:buffer pH 5.3; 0,2 % TMZ
LCP MO:buffer pH 5.3; 0,2 % TMZ + 0,2 % DOX
1.0
0.8
0.6
0.4
0.2
0.0
I/I0
0
20
40
60
80
100
120
140
time / min

D)

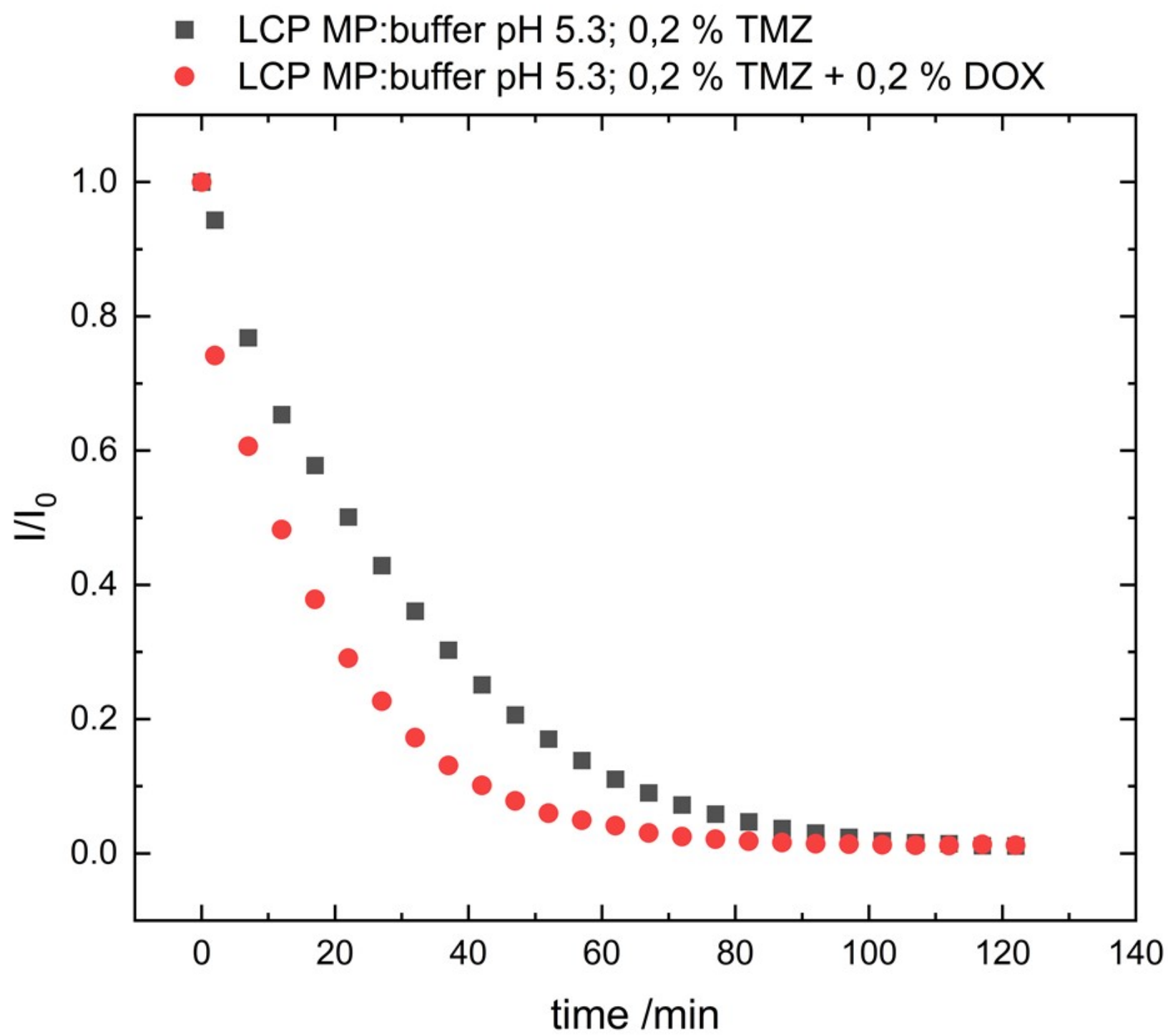
LCP MP:buffer pH 5.3; 0,2 % TMZ
LCP MP:buffer pH 5.3; 0,2 % TMZ + 0,2 % DOX
1.0
0.8
0.6
0.4
0.2
0.0
I/I0
0
20
40
60
80
100
120
140
time /min

Fig. S2 Dose-response relationship between cubosome concentration and cell viability in cells exposed to empty cubosomes.

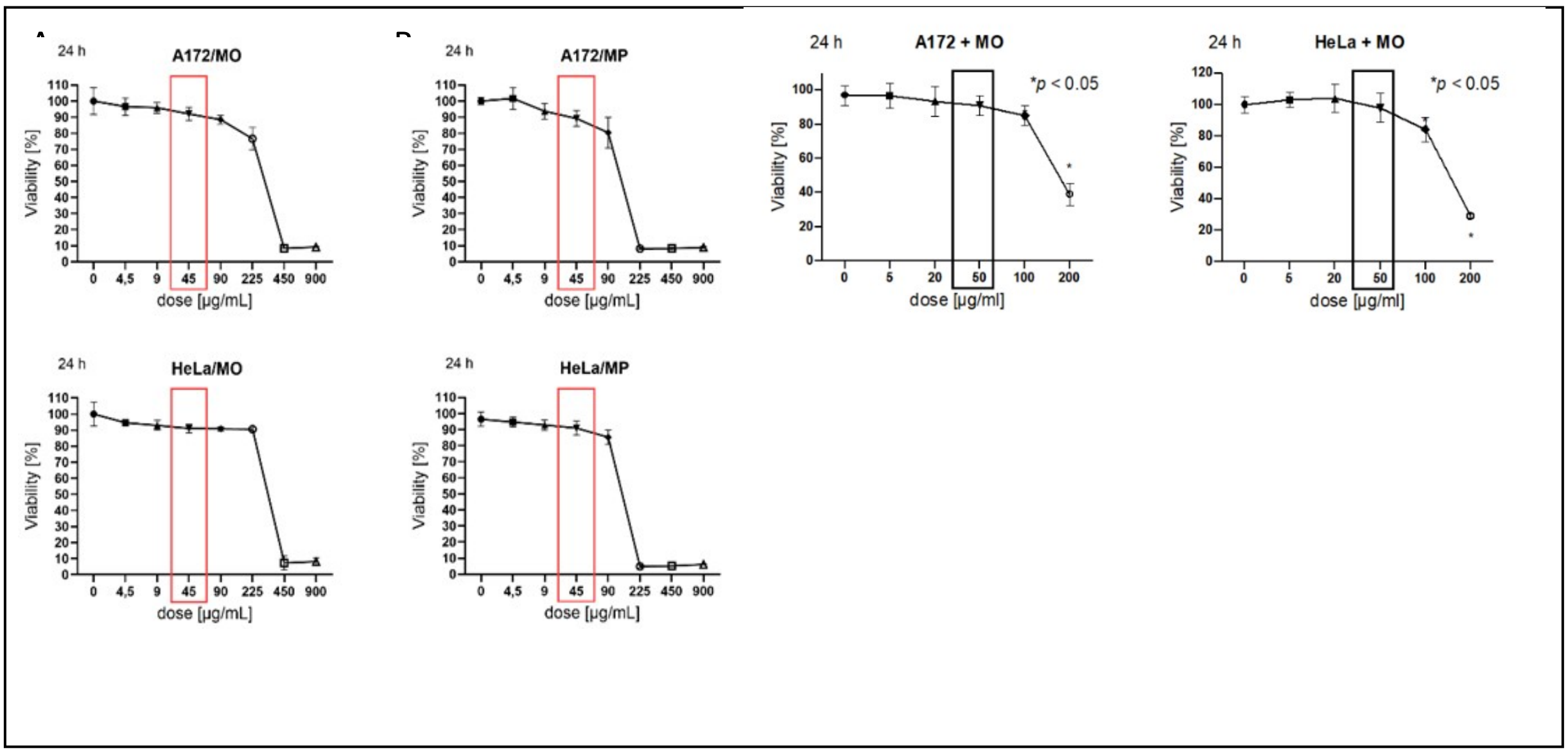

Figures A and B *shows dose-response relationship between cubosomes concentration and cell viability 24 hours after treatment. Cell viability of A172 and HeLa (upper and lower panel, respectively) was determined after exposure to increasing concentrations of the MO and/or MP cubosomes. Statistical analysis was performed using ordinary One-way ANOVA followed multiple comparison. The statistical analysis was conducted on a sample of n=9. Data are presented as mean ± SD; $p < 0.05$.*

Figure C *present dose-response relationship between cubosomes and cell viability 24 hours after treatment. Cell viability of A172 and HeLa (left and right panel, respectively) was determined after exposure to increasing concentrations of the MO cubosomes.*

*The viability of tested cells was assessed using an MTS-based assay (CellTiter 96 AQueous One Solution Cell Proliferation MTS Assay; Promega, Germany). Briefly, cells were seeded in 96-well plates at a density of 4 × 10^3 cells per well in 100 µL of complete growth medium and incubated overnight. The following day, the medium was supplemented with increasing concentration of MO or MP nanoparticles. Untreated cells served as controls. After 24 h of treatment, 20 µL of MTS reagent was added to each well, and the plates were incubated for an additional 3 h. Absorbance was measured at 490 nm using a Synergy2 microplate reader (BioTek Instruments, USA). Cell viability was expressed as a percentage relative to control cells, which were set at 100%. Statistical analysis was performed using ordinary One-way ANOVA followed multiple comparison. The statistical analysis was conducted on a sample of n > 8. Data are presented as mean ± SD; *$p < 0.05$.*

References:

[1] D.M. Anderson, S.M. Gruner, S. Leibler, Geometrical aspects of the frustration in the cubic phases of lyotropic liquid crystals., Proc. Natl. Acad. Sci. 85 (1988) 5364–5368. https://doi.org/10.1073/pnas.85.15.5364.

[2] D.C. Turner, Z.-G. Wang, S.M. Gruner, D.A. Mannock, R.N. McElhaney, Structural study of the inverted cubic phases of di-dodecyl alkyl-β-D-glucopyranosyl-rac-glycerol, J. Phys. II. 2 (1992) 2039–2063.

[3] P. Garstecki, R. Hołyst, Scattering Patterns of Self-Assembled Cubic Phases. 2. Analysis of the Experimental Spectra, Langmuir. 18 (2002) 2529–2537. https://doi.org/10.1021/la011299h.
